# Association Between Serum Bicarbonate Levels and Prediabetes and Subclinical Inflammation in Young Healthy Adults: A Cross-sectional Study

**DOI:** 10.2147/DMSO.S402037

**Published:** 2023-04-04

**Authors:** Abeer A Omar, Khadija J Radwani, Maryam K Al Thani, Nadin H Abouzeid, Yousef E Qabeel, Manwa Al Shamari, Tawanda Chivese, Susu M Zughaier

**Affiliations:** 1 College of Medicine, QU Health, Qatar University, Doha, Qatar

**Keywords:** bicarbonate, prediabetes, subclinical inflammation, males, females, Qatar

## Abstract

**Purpose:**

Low bicarbonate, a hallmark of metabolic acidosis is associated with various diseases. This study investigated associations between bicarbonate levels with prediabetes and subclinical inflammation among healthy young adults in Qatar.

**Patients and Methods:**

A cross-sectional study was carried out with 825 participants aged 18–40 years, devoid of any known comorbidities, using data from the Qatar Biobank. For each participant, blood samples were taken for measurements of bicarbonate, prediabetes, and subclinical inflammation biomarkers. Prediabetes was defined using HbA1c between 5.7 and 6.4% and subclinical inflammation was defined using monocyte to high density lipoprotein (HDL) cholesterol ratio (MHR). Associations between bicarbonate levels and the outcomes were analyzed using multivariable linear and logistic regression and then stratified by gender.

**Results:**

A total of 825 participants with mean age 29.2 years (5.9) of which 365 (44.2%) were males. After multivariable logistic regression, each unit increase in serum bicarbonate was associated with a 17% decreased risk of prediabetes (OR: 0.83, 95%CI: 0.70–0.99, *p*=0.034), in males but no association was observed for females. Similarly, after multivariable linear regression, a unit increase in serum bicarbonate was associated with a 0.18 unit decrease in MHR (beta −0.18, 95%CI: −0.29, −0.07, *p*=0.002), again with no association observed in females.

**Conclusion:**

In a healthy young adult population, higher serum bicarbonate levels were inversely associated with both prediabetes and subclinical inflammation in males, but not in females.

## Introduction

Prediabetes or intermediate hyperglycemia is a high-risk state for developing diabetes with annualized conversion rate of 5–10% and is linked with the simultaneous presence of insulin resistance and β-cell dysfunction that begin before the glucose changes are measurable. People with prediabetes are asymptomatic but have increased risk of both microvascular diseases such as nephropathy, retinopathy, and macrovascular disease such as cardiovascular diseases.^1^ Qatar currently is one of the countries with high prevalence of prediabetes and diabetes at 11.9%^2^ and 16.4%,^3^ respectively, and more research is needed to identify risk factors and preventive factors for both diabetes and prediabetes.

There is evidence that low grade metabolic acidosis maybe associated with insulin resistance and diabetes mellitus.^4^ Bicarbonate, produced by the stomach and the pancreas,^5^ serves as the main buffering system that maintains pH in the normal range between 7.35 and 7.45, and is fundamental for the optimum functioning of organs.^6^ Lower levels of bicarbonate are a marker of chronic low-grade metabolic acidosis and have been linked with decreased insulin sensitivity and diabetes mellitus, although findings are not conclusive.^7^,^8^ Higher levels of inflammatory biomarkers (subclinical inflammation) are associated with the development of chronic diseases and increase in mortality.^9^ The elevation of pro-inflammatory proteins is predictive of type 2 diabetes.^10^ In addition, there is growing evidence suggesting that acidosis has profound effects on the host, particularly in immune function, which may explain the inflammation observed in diabetes, renal and cardiovascular disease.^11^ A previous study has shown that lower bicarbonate levels were associated with higher levels of inflammatory markers (C-reactive proteins) in a healthy population.^9^ Apart from overt inflammation, subclinical inflammation has also been observed in people with diabetes.^12^ Several biomarkers such as monocyte to high density cholesterol ratio (MHR), a novel marker, ferritin, and albumin are also known to reflect subclinical inflammation.^12^,^13^ Research on the association between subclinical inflammation and bicarbonate levels remains limited.

Low grade acidosis maybe increasing in prevalence due to diets that consist of highly processed foods, which are consumed by an increasing number of people worldwide. This unvaried diet was shown to induce chronic low-grade metabolic acidosis in healthy adults.^14^ Studies have shown that the dietary acid load is linked with worsening insulin sensitivity and increased risk of developing type 2 diabetes.^15–19^

Previous research has suggested that bicarbonate has a differential effect in athletes, where male athletes respond well to bicarbonate supplements while female athletes do not show an effect.^20^ We hypothesized that a similar effect may be present in the association between bicarbonate and prediabetes. To our knowledge, research is scarce on the effect of bicarbonate on the risk of prediabetes and subclinical inflammation in the Middle East and North African region. Hence, this study aimed to investigate the associations between bicarbonate levels and prediabetes and subclinical inflammation among healthy young adults in Qatar followed by investigating the same associations in males and females separately. Further, we sought to investigate the associations between bicarbonate and other markers of inflammation and insulin resistance in males and females, separately.

## Materials and Methods

This study is a cross-sectional study of participants, aged between 18 and 40 years without any known diseases, from the Qatar Biobank (QBB) whose data were collected between 2012 and 2017.^21^ For unfunded projects, the QBB gives a maximum number of 1,000 participants. A total of 874 participants were initially included but participants with comorbidities such as cardiovascular disease, diabetes (HbA1c ≥6.5), hypertension, morbid obesity (BMI >40), cancer and any kidney diseases were excluded leaving a total of 825 participants. For each participant, a questionnaire was administered, anthropometry measured, and blood samples were taken for the assessment of bicarbonate and measures of prediabetes and subclinical inflammation.

### Variable Measurements

All variables used in this study were collected using Qatar Biobank established protocol.^22^ Data collected included sociodemographic data and past medical history, anthropometry, blood pressure and laboratory assessments of clinical markers of interest. Laboratory assessments were measured at Hamad Medical Corporation (HMC) laboratories, Department of Laboratory Medicine, and Pathology (DLMP) that holds College of American Pathologist (CAP) accreditation.

#### Exposure – Bicarbonate

Bicarbonate (HCO3^−^) levels were measured for each participant from serum in (mmol/L) having a normal range of 22–28 mmol/L.^23^ Bicarbonate and lipids profiles including HDL-c were measured using the microvolume automated Roche Cobas 6000 biochemical analyser.

#### Outcomes – Prediabetes and Subclinical Inflammation

For each participant HbA1c was measured from whole blood using latex agglutination inhibition with spectrophotometry (Siemens DCA vantage device). A normal level was defined as HbA1c <5.7 and prediabetes as HbA1c of (5.7–6.4), using the prediabetes criteria of the American Diabetes Association.^24^ Other markers of glycemic control assessed were fasting blood glucose and c-peptide. We also assessed insulin resistance defined using a homeostatic model of insulin resistance (HOMA-IR). The Oxford University calculator was used to calculate (HOMA-IR2)^25^ using C-peptide as it is more stable than insulin.^26^

Complete blood count and WBC differential was performed at the same laboratories using microcuvette technology using HemoCue WBC DIFF analyser. WBC differential counts included monocytes percentage. The monocyte percentage to HDL ratio (MHR) level, calculated by dividing monocyte percentage on HDL-c concentration, was used as a marker of subclinical inflammation and oxidative stress to assess the association between low levels of HCO3^−^ and inflammation.^27^ Other markers of inflammation assessed were albumin, ferritin.

### Statistical Analysis

Normality of variables distribution was assessed by generating histograms. For normally distributed variables, means (SD) while median (IQR) was reported for abnormally distributed variables. For categorical variables, frequency tables were used to report frequency and percentages. Box plots were used to compare bicarbonate levels in both genders. For prediabetes, we categorized HbA1c. Linear regression was used to compute unadjusted beta coefficients for the association between bicarbonate and MHR. Logistic regression was used to compute unadjusted odds ratios (OR) for the association between bicarbonate and the following categorical outcome: prediabetes by HbA1c and for insulin resistance, we categorized HOMA-IR according to its upper quartile (75th percentile), with values above 75th percentile (a cut-off of 1.89) considered as high.

Multivariable linear regression was used to compute adjusted beta coefficients for the association between bicarbonate and MHR. Multivariable logistic regression was used to compute adjusted ORs for the association between bicarbonate and prediabetes by HbA1c. In each model, we adjusted age and BMI as confounders. Age was categorized into two categories (18–29) and (30–40). BMI was further categorized into normal <25, overweight 25–29.9, and obese ≥30. Stratified analysis by gender was performed for all the outcomes to test for possible effect modification. Exact *p*-values were reported, and 95%CIs reported for ORs and beta coefficients. We used residual vs predictor plots to assess for homoscedasticity in linear regressions and the link test was used to check for model specification. To investigate the possible cut-off point for bicarbonate associated with prediabetes, we used the Liu method,^28^ which tries to find the cut-off point that corresponds to the maximum of the product of the sensitivity and specificity of a test. In Stata, this was done using the “cutpt” module. All analyses were carried out using Stata statistical software package 16 (STATA 16), (Stata Corp, College Station, TX, USA).

## Results

### Baseline Characteristics

**Figure 1:**
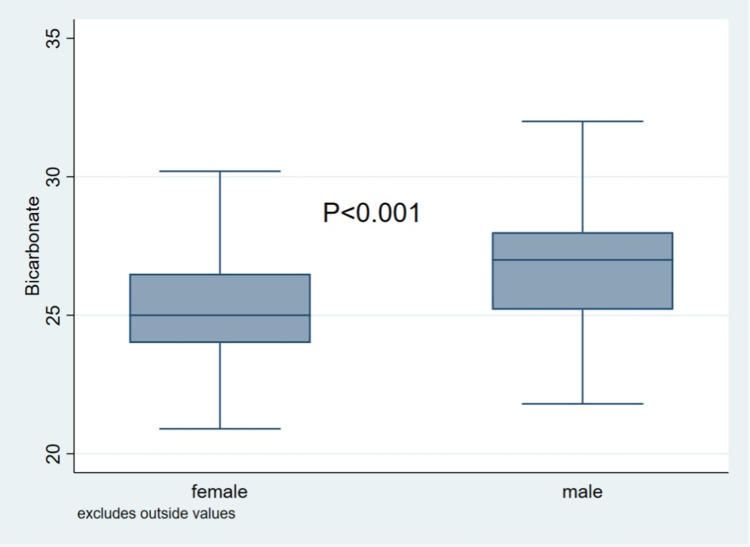
Box plot of serum bicarbonate levels (mmol/L) (females n=460, males n=365).

This cohort comprised of 825 young Qatari participants. Male participants were 365 (44.2%) whereas female participants were 460 (55.8%). In male participants, the mean age was 29.8 years, the mean bicarbonate 26.7 mmol/L, mean HbA1c was 5.2 and mean MHR was 6.7. In female participants the mean age was 28.8, mean bicarbonate was 25.2 mmol/L, mean HbA1c was 5.2 and mean MHR was 4.7 (Table 1). Males had significantly higher mean age, BMI, MHR, bicarbonate, and HOMA-IR, compared to females (Table 1, Figure 1).

**Table 1:** Baseline Characteristics of Included Participants

Characteristics	n (%) or Mean (SD)		*p*value
	Males	Females	
Totals	365 (44.2%)	460 (55.8%)	
Age, years	29.8 (5.9)	28.8 (5.9)	0.011
Age categories			
18–29 years	117 (48.5%)	263 (57.2%)	0.013
30–40 years	188 (51.5%)	197 (42.8%)	
BMI (kg/m^2^)	28.1 (5.9)	27.4 (6.4)	0.104
BMI category			
Normal <25 kg/m^2^	115 (31.5%)	180 (39.1%)	0.007
Overweight 25–29.9 kg/m^2^	146 (40%)	137 (29.8%)	
Obese ≥30 kg/m2	104 (28.5%)	143 (31.1%)	
HbA1c, (%)	5.2 (0.4)	5.2 (0.3)	0.318
HbA1C category			
Normoglycemic	327 (91.1%)	418 (92.9%)	0.318
Prediabetes	32 (8.9%)	32 (7.1%)	
HOMA-IR	1.65 (0.83)	1.51 (0.93)	0.025
HOMA-IR category			
Normal	277 (78.3)	322 (71.7)	0.035
Insulin resistance	77 (21.8)	127 (28.3)	
MHR	6.7 (2.4)	4.7 (1.7)	<0.001
Bicarbonate, (mmol/L)	26.7 (2.2)	25.2 (2.0)	<0.001

### Associations between Bicarbonate Levels and Prediabetes

**Figure 2:**
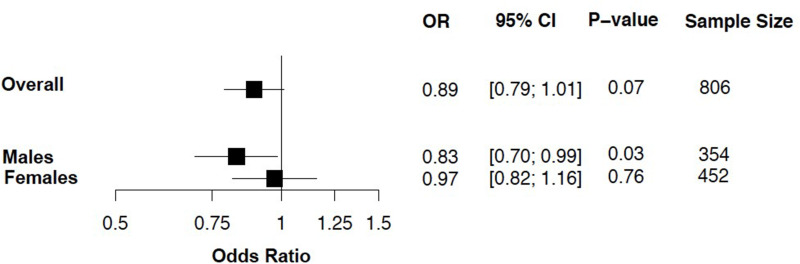
Forest plot showing association between bicarbonate and prediabetes by gender stratification. Adjusted for age and BMI.

The proportion of participants with prediabetes was slightly higher in males (8.9%) in males than in females (7.1%) (Table 1). After multivariable logistic regression, bicarbonate was associated with a 17% decreased risk of prediabetes in males (OR: 0.83, 95%CI: 0.70, 0.99, *p*=0.034). There was no significant association between bicarbonate and prediabetes in females (Figure 2). The same pattern (ie the inverse association between bicarbonate in males and no effect in females) was observed when the analysis was repeated in both genders separately in the age groups of 18–29 years and 30–40 years (Supplementary Tables 1–4). Further analysis showed that the cut-off point for bicarbonate for increased odds of prediabetes was 26.75 in males. This cut-off point had a sensitivity of 54%, a specificity of 63% and an area under the receiver operating curve of 0.59, indicating that bicarbonate alone is insufficient in diagnosing or screening for prediabetes. The cut-off was not investigated in females as there were no clinical and statistically significant associations between bicarbonate and prediabetes in females.

### Associations between Bicarbonate Levels and Subclinical Inflammation

**Figure 3:**
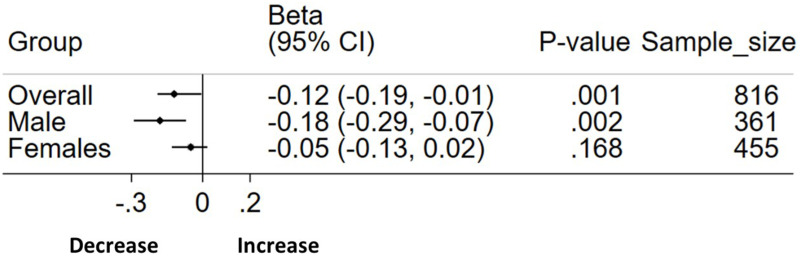
Forest plot showing association between bicarbonate levels and subclinical inflammation marker MHR using multivariable linear regression by gender stratification. Adjusted for age and BMI.

After multivariable linear regression, there was an inverse association between bicarbonate and MHR in both genders (beta −0.12, 95%CI: −0.18, −0.05, *p*=0.001). However, after stratification, the association was stronger in males, where a unit mmol/L increase in bicarbonate was significantly associated with 0.18 decrease in MHR (beta −0.18, 95%CI −0.29, −0.07, *p*=002) but no significant association was observed in females (Figure 3). In the age groups, there was no association between bicarbonate in both males and females aged 18–29 years (Supplementary Tables 5 and 6), but bicarbonate appeared to be protective against subclinical inflammation (higher MHR) in both males and females aged 30–40 years, again with a stronger effect in males (Supplementary Tables 7 and 8).

### Associations between Bicarbonate Levels and Other Markers of Inflammation and Insulin Resistance

In secondary analysis, we observed no association between bicarbonate and ferritin in both genders (Supplementary Tables 9 and 10). However, bicarbonate showed a strong association with higher levels of albumin in males (Supplementary Table 11) and a weaker and nonstatistically significant association with an increase in albumin in females (Supplementary Table 12). A consistent pattern was also observed for HOMA-IR, with bicarbonate showing an inverse association with an increase in HOMA-IR in males but no association in females (Supplementary Tables 13 and 14). However, when HOMA-IR was categorized, there was no association with bicarbonate in both genders (Supplementary Tables 15 and 16). This was also the case with c-peptide (Supplementary Tables 17 and 18) and glucose (Supplementary Tables 19 and 20).

## Discussion

In this study, we found that higher levels of serum bicarbonate were inversely associated with both prediabetes and subclinical inflammation in young males but not in young females suggesting a protective effect of higher levels of bicarbonate in males. We found that males had 17% decreased odds of developing prediabetes with every 1 mmol/L increase in bicarbonate levels. Conversely, there were no significant associations in females. Not many studies have been performed to assess the gender stratified associations of bicarbonate and prediabetes. Findings from one nested case control study^4^ among nurses aged 30–55 years showed that higher bicarbonate levels were associated with lower odds of getting type 2 diabetes. Women with plasma bicarbonate above the median level had lower odds of diabetes (OR: 0.76, 95%CI: 0.60–0.96).^4^ However, this study had not included males, and the endpoint was diabetes, different from our endpoint of prediabetes. Another difference between the two studies is that we included only healthy participants, without any known comorbidities. The mechanism by which bicarbonate exerts a protective effect in either diabetes or prediabetes is not completely understood. In the present study, it is also not clear why there is a differential effect, stronger in men and very weak in women. However, like our study, gender differences have been observed in one study before, in response to bicarbonate supplementation, where male athletes showed strong beneficial responses in several performance indicators while there were no observed benefits in women athletes.^20^ We also observed similar gender-stratified associations between bicarbonate and HOMA-IR but none with the categorized insulin resistance, c-peptide, and glucose. Various factors may explain why bicarbonate decreased the odds of prediabetes in males only. A possible explanation could be related to the differences in pancreatic fluid excretion, which is involved in secreting bicarbonate between the two genders. Males have greater output volume of pancreatic bicarbonate fluid 20% more than females and this is due to the body size where males have greater body size compared to females not necessarily that there is a gender difference in the pancreas itself.^29^ Therefore, the higher levels of bicarbonate could be more protective in males compared to females since lower bicarbonate a marker of chronic low grade metabolic acidosis is independently associated with decreased insulin sensitivity and higher levels of inflammatory markers.^7^,^9^

We found an (inverse) association between higher bicarbonate levels and two markers of subclinical inflammation, again with the association being observed in males but not in females. In males, a decrease in 0.18 in MHR was observed for every 1 mmol/L increase in bicarbonate, and similar associations observed for albumin with a beta of −0.26 in males. For females, both MHR and albumin showed diminished nonsignificant associations with bicarbonate. We found no studies which investigated the association between bicarbonate and these subclinical inflammation markers, especially MHR, due to its novel nature. Again, it is not clear why bicarbonate had a stronger and protective effect in males than in females in our study. The previously mentioned research suggested that males may respond more, physiologically, to bicarbonate supplementation, compared to females, and this may explain some of our observed findings for subclinical inflammation too.^20^ This could possibly stem from physiological differences like females have smaller type II muscle fibers compared to men which rely mainly on glycolytic energy system. Males have shown to have greater glycolytic capacity.^20^ The glycolytic state is linked to increase lactate and hydrogen ions through anaerobic respiration which results in decreased pH. Bicarbonate then increases and acts as a buffer through the process called bicarbonate loading.^30^ Therefore, in this study comprising of young healthy adults, males could be responding more to an alkaline diet or supplements than females which may explain why males had decreased odds of prediabetes and decreased risk of subclinical inflammation. Alternatively, estradiol, although not well proven, is believed to stimulate duodenal bicarbonate secretion through intracellular calcium mobilization and stimulation of Cl^−^/HCO_3_^−^ anion exchange.^31^ One study showed that estrogen receptors are expressed in the duodenal mucosa which stimulates duodenal bicarbonate secretion explaining the lower prevalence of duodenal ulcers in premenopausal women compared to men.^32^

Bicarbonate has generally been disregarded as therapeutic use in treating critically ill people with severe metabolic acidosis. According to a systemic review, limited benefits were seen in using bicarbonate as therapy to severe acidosis in ICU, but may offer effective outcomes in carefully selected populations.^33^

Whether bicarbonate supplementation reduces the risk of prediabetes or insulin sensitivity is still not clear, as randomized controlled trials have yielded conflicting results. One study showed bicarbonate supplementation improves insulin resistance (HOMA index) in individuals with chronic kidney disease and type 2 diabetes^34^ while a three-month randomized control trial performed in nondiabetic older adults 50 years and above, who used bicarbonate supplementation to improve bone function, showed no improvement in insulin sensitivity or glucose control.^35^ These findings suggest a need for more research.

A strength of this study is it provides tentative findings on a research area that is under researched, and that we have carried out a robust and exhaustive analysis in a sample of healthy adults. This study has several limitations. This study was a cross-sectional study thus, no temporality between bicarbonate and various markers could be inferred and hence no inferences about causality can be made from the study. Data on possible confounders such as diets or supplements of the participants were not available. There were no data for arterial pH, venous pH, and urinary pH, which limited the ability to categorize the acidity status of participants. However, since this was a healthy cohort devoid of any known comorbidities, it was unlikely to find participants with clinical metabolic acidosis.

## Conclusion

In a healthy young population, higher serum bicarbonate levels are inversely associated with both prediabetes and subclinical inflammation in males but not in females. Our findings could suggest a possible protective effect of higher serum bicarbonate levels against metabolic diseases such as prediabetes and subclinical inflammation in young men but not in young women. Further research is required to explore these findings in other population studies, to explain the mechanism by which this occurs, and to explore the effect of increased alkaline diet or oral supplemental bicarbonate on inflammatory biomarkers, prediabetes, and type 2 diabetes mellitus.

## Data Sharing Statement

The data used in the current study are obtained under Material Transfer Agreement from Qatar Biobank repository in Doha, Qatar (https://www.qatarbiobank.org.qa/) therefore are not publicly available.
